# Evaluation of Thyroid Function in Patients Hospitalized for Acute Heart Failure

**DOI:** 10.1155/2021/6616681

**Published:** 2021-03-31

**Authors:** Ana Rita Leite, João Sérgio Neves, Marta Borges-Canha, Catarina Vale, Madalena von Hafe, Davide Carvalho, Adelino Leite-Moreira

**Affiliations:** ^1^Departamento de Cirurgia e Fisiologia, Unidade de Investigação Cardiovascular, Faculdade de Medicina da Universidade do Porto, Porto, Portugal; ^2^Department of Endocrinology, Diabetes and Metabolism, Centro Hospitalar Universitário de São João, Faculdade de Medicina da Universidade do Porto, Porto, Portugal; ^3^Instituto de Investigação e Inovação em Saúde (i3S), Universidade do Porto, Porto, Portugal

## Abstract

**Background:**

Thyroid hormones (TH) are crucial for cardiovascular homeostasis. Recent evidence suggests that acute cardiovascular conditions, particularly acute heart failure (AHF), significantly impair the thyroid axis. Our aim was to evaluate the association of thyroid function with cardiovascular parameters and short- and long-term clinical outcomes in AHF patients.

**Methods:**

We performed a single-centre retrospective cohort study including patients hospitalized for AHF between January 2012 and December 2017. We used linear, logistic, and Cox proportional hazard regression models to analyse the association of thyroid-stimulating hormone (TSH) and free thyroxine (FT4) with inpatient cardiovascular parameters, in-hospital mortality, short-term adverse clinical outcomes, and long-term mortality. Two models were used: (1) unadjusted, and (2) adjusted for age and sex.

**Results:**

Of the 235 patients included, 59% were female, and the mean age was 77.5 (SD 10.4) years. In the adjusted model, diastolic blood pressure was positively associated with TSH [*β* = 2.68 (0.27 to 5.09); *p* = 0.030]; left ventricle ejection fraction (LVEF) was negatively associated with FT4 [*β* = -24.85 (-47.87 to -1.82); *p* = 0.035]; and a nonsignificant trend for a positive association was found between 30-day all-cause mortality and FT4 [OR = 3.40 (0.90 to 12.83); *p* = 0.071]. Among euthyroid participants, higher FT4 levels were significantly associated with a higher odds of 30-day all-cause death [OR = 4.40 (1.06 to 18.16); *p* = 0.041]. Neither TSH nor FT4 levels were relevant predictors of long-term mortality in the adjusted model.

**Conclusions:**

Thyroid function in AHF patients is associated with blood pressure and LVEF during hospitalization. FT4 might be useful as a biomarker of short-term adverse outcomes in these patients.

## 1. Introduction

Thyroid hormones (TH) have several important functions in the maintenance of cardiovascular homeostasis that have been extensively demonstrated in numerous experimental and clinical studies [1, 2]. TH strongly affect cardiac electrophysiology and contractility, vascular resistance, and lipid metabolism. Therefore, clinical thyroid dysfunction is known to be responsible for inducing or exacerbating several cardiovascular diseases, such as atrial or ventricular arrhythmias, hypertension, dyslipidaemia, atherosclerosis, and heart failure, increasing cardiovascular morbidity and mortality [1].

In the last decades, there has been a growing body of evidence supporting subclinical thyroid dysfunction as a contributor to the increase of cardiovascular risk [3]. Subclinical hypothyroidism, defined biochemically as elevated serum thyroid-stimulating hormone (TSH) levels and normal free thyroxine (FT4) and free triiodothyronine (FT3) levels, has been associated with worsened left ventricular diastolic function [4]; endothelial dysfunction [5]; and higher levels of LDL, total cholesterol and triglycerides [6], particularly when TSH > 10 mIU/L. On the other hand, subclinical hyperthyroidism, characterized by low serum TSH and normal FT4 and FT3 levels, has been associated with a higher risk of developing arrhythmias, such as atrial fibrillation, endothelial dysfunction, and increased thrombogenicity [7, 8]. Even in euthyroid individuals, high-normal T4 levels are associated with an increased risk of incident atrial fibrillation [9], heart failure [10], and sudden cardiac death [11].

The magnitude of the effects of thyroid dysfunction depends on the underlying cardiovascular condition of the patient. Indeed, patients with heart failure may have a lower tolerance to subtle changes in thyroid function [12]. In this population, mildly elevated TSH levels are consistently associated with more severe heart failure and are predictive of a worse clinical evolution [12–15].

In addition to the impact of TH on the cardiovascular system, both acute and chronic cardiovascular diseases modulate the set point of the hypothalamus-pituitary-thyroid axis. Several theories have tried to explain the underlying cause of these changes [16]. Low-T3 syndrome, defined as the presence of low FT3 levels with normal levels of TSH and normal or high-normal FT4 levels [17], has been well described in patients with critical illnesses, particularly in hospitalized patients [18]. This syndrome is common in patients with acute [19] and chronic heart failure [20, 21] and has been independently associated with a higher risk of mortality [22, 23].

Acute heart failure (AHF) is the most common cause of unplanned hospital admission in patients older than 65 years [24]. Hospitalization of patients with preexisting heart failure is a strong prognostic predictor of increased mortality [25]. Thus, changes in thyroid function during AHF may substantially contribute to cardiovascular morbidity and mortality of these patients. However, studies evaluating the prognostic relevance of TH in patients with AHF are sparse and have shown conflicting results [26–32]. Therefore, we aimed to evaluate the association of thyroid function with clinical, analytical, and echocardiographic parameters during AHF hospitalization as well as the predictive value of TH in these patients.

## 2. Materials and Methods

### 2.1. Study Design and Participants

We performed a retrospective cohort study evaluating hospitalized patients aged 18 years or older with the primary diagnosis of AHF, as determined by a specialist in our institution, between January 2012 and December 2017. AHF was defined as a rapid onset or worsening of symptoms or signs of heart failure requiring urgent hospital admission for evaluation and treatment [33]. From the 494 firstly screened, we excluded patients who were missing TSH measurements within the first 72 hours of hospitalization (*n* = 193) or treated with medications that could interfere with thyroid function assessment, namely, levothyroxine (*n* = 38), amiodarone (*n* = 21), antithyroid medications (methimazole or propylthiouracil, *n* = 5), or lithium (*n* = 2). Two hundred and thirty-five patients were included in our analysis.

Our study was approved and monitored by the Ethics Committee of Centro Hospitalar Universitário de São João.

### 2.2. Clinical and Biochemical Parameters Evaluated

We collected patients' demographic, clinical, and analytical information at the time of hospitalization. We recorded vital signs evaluated on admission to the emergency department (heart rate and systolic and diastolic blood pressure). Biochemical parameters measured on admission to the emergency department [serum creatinine, urea, and b-type natriuretic peptide (BNP)] and thyroid function measurements (TSH and FT4) obtained from blood samples within the first 72 hours of hospitalization, were collected from inpatient laboratory databases. Glomerular filtration rate was estimated using Chronic Kidney Disease Epidemiology Collaboration (CKD-EPI) formula, according to KDIGO guidelines on chronic kidney disease (CKD) [34, 35]. Euthyroidism was defined by TSH values within the reference range considered by the laboratory of our centre (0.35 to 4.94 *μ*IU/mL). Left ventricle (LV) ejection fraction and pulmonary arterial systolic pressure (PASP) were obtained from echocardiography performed during hospitalization.

The following medical conditions and comorbidities were collected: history of chronic heart failure (CHF), valvular disease, coronary artery disease, hypertension, atrial fibrillation, diabetes, dyslipidaemia, CKD, chronic obstructive pulmonary disease (COPD), and smoking status. Body mass index (BMI) within 12 months prior to hospitalization was also assessed by reviewing electronic medical records. Information was ascertained based on diagnosis or procedures using relevant *International Classification of Diseases, Ninth Revision* (ICD-9) codes, laboratory results, and records from the episode of hospitalization and ambulatory visits. We considered the absence of registries regarding the aforementioned diagnosis as the absence of disease. Heart failure diagnosis was made by a cardiology or internal medicine specialist and confirmed by the European Society of Cardiology criteria for the diagnosis of heart failure [33]. Heart failure was characterized in terms of LV ejection fraction and NYHA class previous to hospitalization. Coronary artery disease was defined as the occurrence of angina, prior myocardial infarction, or prior coronary revascularization. Diabetes was defined by glycated haemoglobin ≥6.5% during hospitalization, the use of antihyperglycemic drugs, or was determined by medical history [36]. Dyslipidaemia was defined by serum low-density lipoprotein (LDL) cholesterol ≥160 mg/dL, serum high-density lipoprotein (HDL) cholesterol <40 mg/dL, or serum triglycerides ≥200 mg/dL during hospitalization, the use of lipid-lowering agents, or was determined by medical history [37]. History of hypertension, valvular disease, atrial fibrillation, CKD, COPD, and smoking status were determined solely by the patient's medical history.

### 2.3. Follow-Up Data

Patients were followed from the day of admission to the date of death or until October 2019. Patients without complete follow-up were censored at the time of the last known contact with the hospital.

### 2.4. Study Outcomes

The prespecified primary endpoints of the present analysis were as follows: (1) in-hospital death; (2) 30-day postdischarge readmission for AHF; (3) 30-day postdischarge all-cause death; and (4) all-cause mortality and cardiovascular mortality. Cardiovascular death was defined as death attributable to CHF, coronary artery disease, cerebrovascular events, and other cardiovascular causes [38].

### 2.5. Statistical Analysis

Continuous variables are presented as mean (standard deviation, SD) or median (interquartile range, IQR), as appropriate. Categorical variables are presented as percentages. Comparisons between groups were performed using Student's *t*-test.

We used linear regression models unadjusted and adjusted for age and sex to analyse the association of TSH and FT4 with heart rate and blood pressure on admission and inpatient biochemical and echocardiographic parameters. BNP and TSH were analysed as continuous variables after natural logarithmic transformation to normalize their distribution. Logistic regression models unadjusted and adjusted for age and sex were used to evaluate the association of thyroid function with in-hospital mortality, and 30-day postdischarge readmission for AHF and all-cause mortality. The associations of TH and other baseline characteristics measured during hospitalization with all-cause mortality and cardiovascular mortality were assessed using unadjusted and age- and sex-adjusted Cox proportional hazard regression models. These analyses were also performed limiting the cohort to patients with thyroid function within the reference range.

We considered a two-sided *p* value <0.05 statistically significant. Statistical analyses were performed with Stata software, version 14.1 (StataCorp).

## 3. Results

### 3.1. Baseline Population Characteristics

Baseline characteristics of the 235 patients included in the analysis are shown in Table 1. BMI, LV ejection fraction and NYHA class prior to hospitalization were available in 42% (*n* = 98), 86% (*n* = 201), and 66% (*n* = 156) of the cohort, respectively. Echocardiography during hospitalization was performed on 104 patients. LV ejection fraction quantitatively assessed by modified Simpson's rule [39] and PASP were available in 44% (*n* = 46) and 80% of these patients (*n* = 83), respectively. Regarding biochemical data, 83% percent of the patients (*n* = 194) had a measurement of FT4.

The mean age was 77.4 (SD 10.4) years and 59% were women. The median length of stay in hospital was 8 (IQR 5-12) days. Ninety-three percent of the patients had a history of CHF and approximately half of these had an established LV ejection fraction ≥50%. Forty-six percent had coronary artery disease, 78% had hypertension, and 57% had diabetes. The median TSH levels were 1.12 (IQR 0.59–2.11) *μ*IU/mL and the mean FT4 levels in the cohort were 1.14 (SD 0.27) ng/dL. Eighty-five percent (*n* = 200) of the patients were euthyroid on admission.

### 3.2. Association of Thyroid Status with Cardiovascular Function on Admission

Associations of thyroid function with parameters of cardiovascular function measured during hospitalization are shown in Table 2. Mean diastolic blood pressure was positively associated with TSH levels, in univariate [*β* = 2.74 (0.34 to 5.13); *p*=0.025] and multivariate [*β* = 2.68 (0.27 to 5.09); *p*=0.030] regression models. A nonsignificant trend to a positive association was also present after restricting the analysis to euthyroid patients (Table S1). Heart rate and BNP levels were not significantly associated with TH. Regarding echocardiographic parameters, inpatient LV ejection fraction was negatively associated with FT4 levels, even after adjustments for age and sex [*β* = −24.85 (−47.87 to −1.82); *p*=0.035]. This association was lost after considering solely the euthyroid patients (Table S1). No association was found between PASP and TH.

### 3.3. Association of Thyroid Status with Short-Term Clinical Outcomes

Eleven patients (4.7%) died during hospitalization. At 30 days after discharge, 51 (21.7%) had been readmitted for acute decompensation of heart failure and 28 (11.9%) had died. Eighteen (67%) had died from cardiovascular causes.

**Figure 1:**
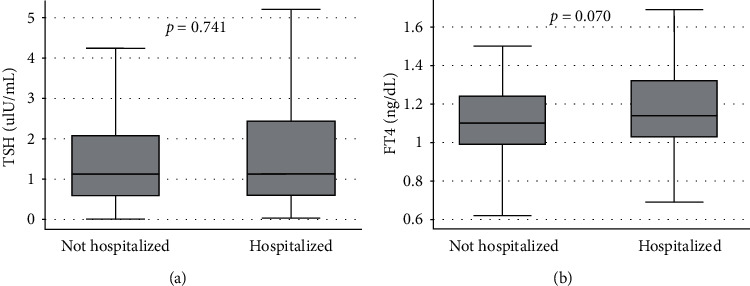
Comparison of (a) TSH and (b) FT4 levels in patients hospitalized and nonhospitalized within 30 days from discharge. TSH: thyroid-stimulating hormone; FT4: free thyroxine.

No association was found between TH and in-hospital death. Patients who were rehospitalized due to heart failure within 30 days of discharge showed a nonsignificant trend for higher levels of FT4 in comparison to patients who were not rehospitalized during this period (*p*=0.07, Figure 1). No difference was perceived regarding TSH levels.

A trend for higher odds of 30-day readmission and all-cause death with higher FT4 levels was demonstrated in the univariate logistic regression analysis (Table 3). Also, higher FT4 levels were significantly associated with higher odds of 30-day cardiovascular death, even after adjustments. After restricting the analysis to patients with thyroid function within the reference range, higher FT4 levels were significantly associated with higher odds of 30-day all-cause and cardiovascular death, in both univariate and multivariate analysis (Table S2).

### 3.4. Association of Inpatient Parameters with Long-Term Clinical Outcomes

The median follow-up period was 349 (IQR 94-1087) days. The overall mortality rate during this period was 66% (*n* = 155). Fifty-two percent (*n* = 80) of these patients died from cardiovascular causes. Associations between parameters measured during hospitalization and long-term clinical outcomes are shown in Table 4.

Neither TSH nor FT4 levels were associated with an increased risk of long-term all-cause mortality. Higher echocardiographic PASP levels were associated with higher all-cause mortality, in both unadjusted [HR = 1.03 (1.02 to 1.05); *p* < 0.001] and adjusted [HR = 1.03 (1.02 to 1.05); *p* < 0.001] models. After restricting the analysis to euthyroid patients, both higher BNP and PASP levels, and lower eGFR levels were associated with a higher risk of all-cause mortality, in the unadjusted and adjusted analysis (Table S3).

There was no significant association between TSH or FT4 levels and cardiovascular mortality. Increased BNP and PASP levels were associated with a higher cardiovascular mortality, even after adjustments [HR = 1.35 (1.08 to 1.70); *p* = 0.009 and HR = 1.04 (1.02 to 1.06); *p* < 0.001, respectively]. Similar results were found after restricting the analysis to euthyroid patients (Table S3).

## 4. Discussion

In a cohort of patients admitted for AHF, thyroid hormone levels during hospitalization were shown to be significantly associated with markers of cardiovascular function, such as blood pressure and ejection fraction. Our study showed a consistent positive association between TSH levels and diastolic blood pressure. On the other hand, FT4 levels were negatively associated with inpatient LV ejection fraction. Also, a positive association between FT4 levels and death within 30 days from discharge was found among patients with thyroid function within the reference range. However, neither TSH nor FT4 levels had shown to be relevant predictors of long-term mortality.

Several studies have demonstrated that the vascular system is an important target of TH action. TH promote vasodilation through increased production of nitric oxide by endothelial cells and relaxation of smooth muscle cells, mainly mediated by T3 [1]. Indeed, overt and subclinical hypothyroidism are associated with diastolic hypertension [1], and even low-normal thyroid function has been associated with higher blood pressure in population-based studies [40, 41]. In agreement with these findings, our results suggest that progressively increased levels of TSH, which suggest a lower thyroid function, are associated with higher diastolic blood pressure in this specific population.

On the other hand, the negative association between FT4 levels and inpatient LV ejection fraction, a marker of systolic function, may appear counterintuitive in light of the role of TH in cardiac function [42, 43]. However, this association appears to be significant only with FT4 levels above the reference range, as the significance was lost after restricting the analysis to euthyroid patients. TH have strong genomic and nongenomic effects in cardiac electrophysiology, and hyperthyroid states are linked to increased supraventricular ectopic activity, particularly in patients with reduced cardiovascular reserve [44]. The arrhythmogenic effects of TH in the high range may thus have contributed to LV dysfunction in the acute phase of heart failure [2].

Our study also suggests that higher FT4 levels may be predictors of short-term clinical events as a trend to a positive association between FT4 and short-term readmission for acute exacerbated heart failure and 30-day all-cause and cardiovascular death was found. In fact, the majority of the patients who died within the first 30 days after discharge died from cardiovascular causes. These associations became stronger after restricting the analysis to euthyroid patients. A higher incidence of arrhythmias as atrial fibrillation with higher FT4 levels may have contributed to the increased short-term morbidity and mortality [9, 45].

Nevertheless, other hypotheses may explain the association between FT4 levels and short-term adverse clinical outcomes, particularly among euthyroid patients. Although both T4 and T3 have biological actions on the cardiovascular system, T3 is considered the more biologically active hormone [46]. Higher FT4 levels in AHF patients may not reflect a higher thyroid function but lower peripheral deiodination of T4 to T3 by type 1 deiodinase (D1) and type 2 deiodinase (D2) [47]. Reduced activity of D1 and D2 is hypothesized to be one of the mechanisms contributing to low-T3 syndrome [48].

Low-T3 syndrome is very likely to be present in a significant portion of this sample [19]. Hepatic congestion in AHF may decrease local D1 activity and consequently FT3 levels [27]. Also, proinflammatory and stress responses may further contribute to decreasing peripheral conversion of T4 [49–52]. Similarly to patients with CHF [23, 53], patients hospitalized for AHF with impairment of T4 to T3 conversion have a worse clinical status [27], as shown by the increased length of hospital stay, intensive care unit admission risk [19], and in-hospital mortality [23, 29, 30].

On the other hand, we did not find TSH and T4 to be independent predictors of long-term cardiovascular and all-cause mortality, in contrast with other inpatient markers of heart failure severity, namely, BNP and PASP levels. Thyroid function measurements during hospitalization reflect not only the baseline thyroid function but also the transient variation of thyroid function during acute illness. While these values predict short-term outcomes in AHF, the absence of long-term predictive value is probably related to the lack of correlation between thyroid function during the acute phase and after stabilization in most patients [32, 54].

Earlier studies evaluating the prognostic value of TH in hospitalized patients with heart failure have shown contradictory results. In some studies, FT3 and total T3 were found to be independent predictors of all-cause and cardiovascular mortality [23, 27, 31, 32, 55]. On the other hand, Hayashi et al. reported TSH levels and subclinical hypothyroidism as better predictors of cardiovascular events and long-term mortality than FT3 and low-T3 syndrome [26]. Such differences may partially be attributed to the heterogeneity in the characteristics of the cohorts and in the blood sampling timing. Studies measuring TH concentration after stabilization of the patients may find a thyroid function fully restored to the baseline [27, 32], in contrast with those evaluating thyroid function within the first days of admission [26, 31]. Nevertheless, FT3 seems to be the most consistent predictor of long-term all-cause and cardiovascular mortality in patients with both acute and chronic heart failure [12, 22, 55].

Some limitations may be identified in the present study. First, this is a single-centre retrospective study with a relatively small sample size. We believe that a larger sample size might have shown more definitive and consistent associations. Given the small sample size, we only adjusted our statistical models for the most consistent confounders of the association between thyroid and cardiac function [32]. TH were not measured in all patients hospitalized for AHF, as the decision to measure them was dependent on the clinician's judgement, introducing a potential confounding by indication. Furthermore, T3 levels were not evaluated in the majority of the patients, which could help us define low-T3 syndrome and better understand the peripheral conversion status. In fact, greater support to our hypothesis, as well as different and more accurate associations, could have been found if this hormone was available for analysis. Nevertheless, TSH and FT4 are clinically considered the most sensitive and robust markers of thyroid function [10, 56]. Lastly, echocardiography was performed in a relatively low proportion of the patients, which may further increase selection bias.

The results of our analysis are clinically relevant as they highlight that variations in the thyroid axis during AHF hospitalization are associated with cardiovascular function and markers of heart failure severity. Although progress in the treatment of CHF during the last decades has considerably reduced the late mortality, patients hospitalized for heart failure continue to have a mortality and rehospitalization rate within 30 to 60 days after discharge of 15% and 30%, respectively [25, 57]. Thus, the identification of biomarkers that can help stratify patients in accordance with their risk for short-term outcomes would be helpful to identify those with an increased need for close monitorization and intensive care. TH measurement might be useful to identify these patients. It is important to keep in mind that TH should be interpreted as biological risk factors rather than direct causal factors. As stated above, the bidirectional interrelation between endocrine and cardiac systems makes it difficult to establish causality.

## 5. Conclusions

In conclusion, we found that higher FT4 levels during hospitalization for AHF are associated with worsened systolic function, as indicated by lower ejection fraction, and with a trend for higher risk of readmission and all-cause death within 30 days of discharge. Our findings suggest that FT4 might be useful as a biomarker of short-term adverse clinical outcomes. Larger prospective studies exploring the prognostic and pathophysiological role of TH in AHF and in the different underlying diseases leading to AHF are needed.

## Figures and Tables

**Table 1:** Clinical characteristics of the patients (*n* = 235).

Age (years), mean (SD)	77.4 (10.4)
Female sex	58.7
BMI, kg/m^2^, mean (SD)	27.1 (5.3)
Length of stay in hospital, median (IQR)	8 (5–12)

**Comorbidities**	
History of heart failure	92.8
Classification	
LVEF < 40%	39.8
LVEF 40-49%	10.9
LVEF ≥ 50%	49.3

NYHA class prior to hospitalization	
I	9.0
II	52.6
III	35.9
IV	2.6
Coronary artery disease	45.7
Valvular disease	43.8
Atrial fibrillation	48.7
Hypertension	77.9
Diabetes mellitus	57.0
Dyslipidaemia	82.1
Chronic kidney disease	45.1
COPD	22.6
Current smoker	10.0

**Vital signs on admission**	
Heart rate (bpm), mean (SD)	90.0 (24.8)
Blood pressure (mmHg), mean (SD)	
Systolic	131.8 (29.2)
Diastolic	73.5 (18.3)

**Inpatient echocardiographic measures**	
LVEF, %, mean (SD)	36.0 (16.4)
PASP, mmHg, mean (SD)	47.3 (17.6)

**Laboratory values on admission**	
TSH, *μ*IU/mL, median (IQR)	1.12 (0.59–2.11)
Free T4, ng/dL, mean (SD)	1.14 (0.27)
eGFR, mL/min/1.73 m^2^, mean (SD)	47.2 (30.9–67.5)
BNP, pg/mL, median (IQR)	920.2 (477.3–1639.0)

Values are presented as percentages, except when indicated. SD: standard deviation; BMI: body mass index; IQR: interquartile range; LVEF: left ventricle ejection fraction; NYHA: New York Heart Association; COPD: chronic obstructive pulmonary disease; PASP: pulmonary arterial systolic pressure; TSH: thyroid-stimulating hormone; T4: thyroxine; eGFR: estimated glomerular filtration rate; BNP: B-type natriuretic peptide.

**Table 2:** Linear association of thyroid status (TSH and FT4) with cardiovascular function during hospitalization.

	TSH (*μ*IU/mL)		FT4 (ng/dL)	
	*β* (95% CI)	*p* value	*β* (95% CI)	*p* value
**Heart rate**, bpm				
Unadjusted	−1.04 (−4.38 to 2.30)	0.539	9.44 (−4.29 to 23.18)	0.177
Model 1^a^	−1.25 (−4.61 to 2.11)	0.465	9.16 (−4.71 to 23.03)	0.194

**Blood pressure**				
**Systolic BP**, mmHg				
Unadjusted	2.15 (−1.71 to 6.01)	0.274	−9.97 (−25.42 to 5.48)	0.205
Model 1^a^	1.88 (−1.95 to 5.72)	0.334	−8.51 (−24.03 to 7.01)	0.281
**Diastolic BP**, mmHg				
Unadjusted	**2.74 (0.34 to 5.13)**	**0.025**	−3.24 (−13.13 to 6.66)	0.519
Model 1^a^	**2.68 (0.27 to 5.09)**	**0.030**	−3.01 (−13.01 to 7.00)	0.554
**BNP,** pg/mL				
Unadjusted model	0.02 (−0.12 to 0.15)	0.828	0.28 (−0.26 to 0.82)	0.305
Model 1^a^	0.05 (−0.08 to 0.19)	0.439	0.19 (−0.34 to 0.72)	0.478

**Echocardiographic measures**				
**LV ejection fraction,** %				
Unadjusted model	−2.16 (−7.30 to 2.98)	0.401	−**29.05 (**−**52.24 to −5.85)**	**0.016**
Model 1^a^	−1.13 (−6.08 to 3.83)	0.649	−**24.85 (**−**47.87 to −1.82)**	**0.035**
**PASP,** mmHg				
Unadjusted model	−0.45 (−4.59 to 3.70)	0.830	12.77 (−4.74 to 30.28)	0.150
Model 1^a^	−0.56 (−4.81 to 3.70)	0.795	13.27 (−4.45 to 31.00)	0.140

The values shown are linear regression coefficients (*β*) and 95% confidence intervals, estimated by uni- and multivariate linear regressions, with TSH or FT4 as the independent variable and heart rate, systolic BP, diastolic BP, BNP, LV ejection fraction, and PASP as dependent variables. TSH and BNP were log-transformed. TSH: thyroid-stimulating hormone; FT4: free thyroxine; CI: confidence interval; BP: blood pressure; BNP: B-type natriuretic peptide; LV: left ventricle; PASP: pulmonary arterial systolic pressure. ^a^Adjusted for age and sex.

**Table 3:** Association of thyroid function (TSH and FT4) with short-term clinical endpoints.

	TSH (*μ*IU/mL)		FT4 (ng/dL)	
	OR (95% CI)	*p* value	OR (95% CI)	*p* value
**In-hospital mortality**				
Unadjusted model	0.88 (0.50 to 1.55)	0.655	0.77 (0.04 to 13.95)	0.860
Model 1^a^	0.95 (0.54 to 1.68)	0.854	0.71 (0.04 to 13.40)	0.818

**Readmission for heart failure within 30 days**				
Unadjusted model	1.05 (0.77 to 1.43)	0.739	2.83 (0.84 to 9.50)	0.093
Model 1^a^	1.08 (0.79 to 1.47)	0.644	2.67 (0.78 to 9.11)	0.118

**All-cause mortality within 30 days**				
Unadjusted model	0.94 (0.64 to 1.37)	0.738	3.30 (0.88 to 12.33)	0.076
Model 1^a^	0.96 (0.65 to 1.40)	0.823	3.40 (0.90 to 12.83)	0.071

**Cardiovascular mortality within 30 days**				
Unadjusted model	0.97 (0.60 to 1.54)	0.886	**6.45 (1.42 to 29.29)**	**0.016**
Model 1^a^	0.97 (0.60 to 1.57)	0.886	**6.58 (1.37 to 31.48)**	**0.018**

The values shown are odds ratios and 95% confidence intervals, estimated by uni- and multivariate logistic regressions, with TSH or FT4 as the independent variable and in-hospital mortality, hospital readmission, and all-cause and cardiovascular mortality within 30 days of discharge as dependent variables. TSH was log-transformed. TSH: thyroid-stimulating hormone; FT4: free thyroxine; OR: odds ratio; CI: confidence interval. ^a^Adjusted for age and sex.

**Table 4:** Association between inpatient parameters and long-term clinical outcomes.

	Unadjusted model		Age- and sex-adjusted model	
	Hazard ratio (95% CI)	*p* value	Hazard ratio (95% CI)	*p* value
**All-cause mortality**				
TSH (*μ*IU/mL)	1.02 (0.88 to 1.19)	0.791	1.03 (0.88 to 1.19)	0.733
FT4 (ng/dL)	1.40 (0.69 to 2.85)	0.350	1.22 (0.59 to 2.52)	0.599
BNP (pg/mL)	1.16 (0.99 to 1.36)	0.074	1.16 (0.99 to 1.37)	0.070
eGFR	**0.99 (0.99 to 1.00)**	**0.015**	0.99 (0.99 to 1.00)	0.105
LV ejection fraction (%)	0.98 (0.96 to 1.01)	0.182	0.98 (0.96 to 1.01)	0.146
PASP (mmHg)	**1.03 (1.02 to 1.05)**	**<0.001**	**1.03 (1.02 to 1.05)**	**<0.001**

**Cardiovascular mortality**				
TSH (*μ*IU/mL)	1.02 (0.82 to 1.26)	0.875	1.02 (0.83 to 1.27)	0.823
FT4 (ng/dL)	1.40 (0.53 to 3.71)	0.501	1.33 (0.49 to 3.58)	0.577
BNP (pg/mL)	**1.35 (1.08 to 1.69)**	**0.007**	**1.35 (1.08 to 1.70)**	**0.009**
eGFR (mL/min/1.73 m^2^)	1.00 (0.99 to 1.01)	0.441	1.00 (0.99 to 1.01)	0.518
LV ejection fraction (%)	0.98 (0.95 to 1.01)	0.268	0.98 (0.94 to 1.01)	0.200
PASP (mmHg)	**1.04 (1.02 to 1.06)**	**<0.001**	**1.04 (1.02 to 1.06)**	**<0.001**

TSH and BNP were log-transformed. CI: confidence interval; TSH: thyroid-stimulating hormone; FT4: free thyroxine; BNP: B-type natriuretic peptide; eGFR: estimated glomerular filtration rate; LV: left ventricle; PASP: pulmonary arterial systolic pressure.

## Data Availability

The datasets generated and/or analysed are available from the corresponding author on reasonable request.

## Supplementary Materials

Supplementary Materials Table S1. The values shown are linear regression coefficients (*β*) and 95% confidence intervals, estimated by uni- and multivariate linear regressions, with TSH or FT4 as the independent variable and heart rate, systolic BP, diastolic BP, BNP, LV ejection fraction, and PASP as dependent variables. Table S2. The values shown are linear regression coefficients (*β*) and 95% confidence intervals, estimated by uni- and multivariate linear regressions, with TSH or FT4 as the independent variable and inhospital mortality, hospital readmission, and all-cause and cardiovascular mortality within 30 days of discharge as dependent variables.

## References

[1] Razvi S., Jabbar A., Pingitore A. (2018). Thyroid hormones and cardiovascular function and diseases. *Journal of the American College of Cardiology*.

[2] Cappola A. R., Desai A. S., Medici M. (2019). Thyroid and cardiovascular disease: research agenda for enhancing knowledge, prevention, and treatment. *Thyroid*.

[3] Biondi B., Cappola A. R., Cooper D. S. (2019). Subclinical hypothyroidism. *JAMA: The Journal of the American Medical Association*.

[4] Chen X., Zhang N., Cai Y., Shi J. (2013). Evaluation of left ventricular diastolic function using tissue Doppler echocardiography and conventional doppler echocardiography in patients with subclinical hypothyroidism aged <60 years: a meta-analysis. *Journal of Cardiology*.

[5] Gao N., Zhang W., Zhang Y.-Z., Yang Q., Chen S.-H. (2013). Carotid intima-media thickness in patients with subclinical hypothyroidism: a meta-analysis. *Atherosclerosis*.

[6] Liu X. L., He S., Zhang S. F. (2014). Alteration of lipid profile in subclinical hypothyroidism: a meta-analysis. *Medical Science Monitor*.

[7] Cappola A. R., Fried L. P., Arnold A. M. (2006). Thyroid status, cardiovascular risk, and mortality in older adults. *JAMA: The Journal of the American Medical Association*.

[8] Popławska-Kita A., Siewko K., Telejko B. (2013). The changes in the endothelial function and haemostatic and inflammatory parameters in subclinical and overt hyperthyroidism. *International Journal of Endocrinology*.

[9] Baumgartner C., Da Costa B. R., Collet T.-H. (2017). Thyroid function within the normal range, subclinical hypothyroidism, and the risk of atrial fibrillation. *Circulation*.

[10] Cappola A. R., Arnold A. M., Wulczyn K., Carlson M., Robbins J., Psaty B. M. (2015). Thyroid function in the euthyroid range and adverse outcomes in older adults. *The Journal of Clinical Endocrinology & Metabolism*.

[11] Chaker L., Van Den Berg M. E., Niemeijer M. N. (2016). Thyroid function and sudden cardiac death. *Circulation*.

[12] Kannan L., Shaw P. A., Morley M. P. (2018). Thyroid dysfunction in heart failure and cardiovascular outcomes. *Circulation: Heart Failure*.

[13] Chen S., Shauer A., Zwas D. R., Lotan C., Keren A., Gotsman I. (2014). The effect of thyroid function on clinical outcome in patients with heart failure. *European Journal of Heart Failure*.

[14] Mitchell J. E., Hellkamp A. S., Mark D. B. (2013). Thyroid function in heart failure and impact on mortality. *JACC: Heart Failure*.

[15] Yang G., Wang Y., Ma A., Wang T. (2019). Subclinical thyroid dysfunction is associated with adverse prognosis in heart failure patients with reduced ejection fraction. *BMC Cardiovascular Disorders*.

[16] Vale C., Neves J. S., Von Hafe M., Borges-Canha M., Leite-Moreira A. (2019). The role of thyroid hormones in heart failure. *Cardiovascular Drugs and Therapy*.

[17] Warner M. H., Beckett G. J. (2010). Mechanisms behind the non-thyroidal illness syndrome: an update. *Journal of Endocrinology*.

[18] Fliers E., Bianco A. C., Langouche L., Boelen A. (2015). Thyroid function in critically ill patients. *The Lancet Diabetes & Endocrinology*.

[19] Rothberger G. D., Gadhvi S., Michelakis N., Kumar A., Calixte R., Shapiro L. E. (2017). Usefulness of serum triiodothyronine (T3) to predict outcomes in patients hospitalized with acute heart failure. *The American Journal of Cardiology*.

[20] Ascheim D. D., Hryniewicz K. (2004). Thyroid hormone metabolism in patients with congestive heart failure: the low triiodothyronine state. *Thyroid*.

[21] Galli E., Pingitore A., Iervasi G. (2010). The role of thyroid hormone in the pathophysiology of heart failure: clinical evidence. *Heart Failure Reviews*.

[22] Passino C., Pingitore A., Landi P. (2009). Prognostic value of combined measurement of brain natriuretic peptide and triiodothyronine in heart failure. *Journal of Cardiac Failure*.

[23] Iervasi G., Pingitore A., Landi P. (2003). Low-T3 syndrome. *Circulation*.

[24] Mebazaa A., Yilmaz M. B., Levy P. (2015). Recommendations on pre-hospital & early hospital management of acute heart failure: a consensus paper from the heart failure association of the European society of cardiology, the European society of emergency medicine and the society of academic emergency medicine. *European Journal of Heart Failure*.

[25] Gheorghiade M., Vaduganathan M., Fonarow G. C., Bonow R. O. (2013). Rehospitalization for heart failure. *Journal of the American College of Cardiology*.

[26] Hayashi T., Hasegawa T., Kanzaki H. (2016). Subclinical hypothyroidism is an independent predictor of adverse cardiovascular outcomes in patients with acute decompensated heart failure. *ESC Heart Failure*.

[27] Sato Y., Yoshihisa A., Kimishima Y. (2019). Low T3 syndrome is associated with high mortality in hospitalized patients with heart failure. *Journal of Cardiac Failure*.

[28] Sato Y., Yoshihisa A., Kimishima Y. (2018). Subclinical hypothyroidism is associated with adverse prognosis in heart failure patients. *Canadian Journal of Cardiology*.

[29] Okayama D., Minami Y., Kataoka S., Shiga T., Hagiwara N. (2015). Thyroid function on admission and outcome in patients hospitalized for acute decompensated heart failure. *Journal of Cardiology*.

[30] Asai K., Shirakabe A., Kiuchi K. (2020). Relation of low triiodothyronine syndrome associated with aging and malnutrition to adverse outcome in patients with acute heart failure. *The American Journal of Cardiology*.

[31] Chuang C.-P., Jong Y.-S., Wu C.-Y., Lo H.-M. (2014). Impact of triiodothyronine and N-terminal pro-B-type natriuretic peptide on the long-term survival of critically ill patients with acute heart failure. *The American Journal of Cardiology*.

[32] Frey A., Kroiss M., Berliner D. (2013). Prognostic impact of subclinical thyroid dysfunction in heart failure. *International Journal of Cardiology*.

[33] Ponikowski P., Voors A. A., Anker S. D. (2016). 2016 ESC Guidelines for the diagnosis and treatment of acute and chronic heart failure. *European Journal of Heart Failure*.

[34] Levey A. S., Stevens L. A., Schmid C. H. (2009). A new equation to estimate glomerular filtration rate. *Annals of Internal Medicine*.

[35] Kidney Disease: Improving Global Outcomes (KDIGO) CKD Work Group (2013). KDIGO clinical practice guideline for the evaluation and management of chronic kidney disease. *Kidney International Supplements*.

[36] American Diabetes Association (2020). 2. Classification and diagnosis of diabetes: standards of medical care in diabetes—2020. *Diabetes Care*.

[37] Cleeman J. I. (2001). Executive summary of the third report of the National Cholesterol Education Program (NCEP) expert panel on detection, evaluation, and treatment of high blood cholesterol in adults (adult treatment panel III). *JAMA: The Journal of the American Medical Association*.

[38] Leening M. J. G., Kavousi M., Heeringa J. (2012). Methods of data collection and definitions of cardiac outcomes in the Rotterdam Study. *European Journal of Epidemiology*.

[39] Lang R. M., Badano L. P., Mor-Avi V. (2015). Recommendations for cardiac chamber quantification by echocardiography in adults: an update from the American Society of Echocardiography and the European Association of Cardiovascular Imaging. *Journal of the American Society of Echocardiography*.

[40] Åsvold B. O., Bjøro T., Nilsen T. I. L., Vatten L. J. (2007). Association between blood pressure and serum thyroid-stimulating hormone concentration within the reference range: a population-based study. *Journal of Clinical Endocrinology & Metabolism*.

[41] Åsvold B. O., Bjøro T., Vatten L. J. (2013). Associations of TSH levels within the reference range with future blood pressure and lipid concentrations: 11-year follow-up of the HUNT study. *European Journal of Endocrinology*.

[42] Hoit B. D., Khoury S. F., Shao Y., Gabel M., Liggett S. B., Walsh R. A. (1997). Effects of thyroid hormone on cardiac *β*-adrenergic responsiveness in conscious baboons. *Circulation*.

[43] Holt E., Sjaastad I., Lunde P. K., Christensen G., Sejersted O. M. (1999). Thyroid hormone control of contraction and the Ca2+-ATPase/phospholamban complex in adult rat ventricular myocytes. *Journal of Molecular and Cellular Cardiology*.

[44] Bielecka-Dabrowa A., Mikhailidis D. P., Rysz J., Banach M. (2009). The mechanisms of atrial fibrillation in hyperthyroidism. *Thyroid Research*.

[45] Chaker L., Heeringa J., Dehghan A. (2015). Normal thyroid function and the risk of atrial fibrillation: the Rotterdam study. *The Journal of Clinical Endocrinology & Metabolism*.

[46] Laurberg P. (1984). Mechanisms governing the relative proportions of thyroxine and 3,5,3’-triiodothyronine in thyroid secretion. *Metabolism*.

[47] Bianco A. C., Salvatore D., Gereben B., Berry M. J., Larsen P. R. (2002). Biochemistry, cellular and molecular biology, and physiological roles of the iodothyronine selenodeiodinases. *Endocrine Reviews*.

[48] Peeters R. P., Wouters P. J., Kaptein E., Van Toor H., Visser T. J., Van den Berghe G. (2003). Reduced activation and increased inactivation of thyroid hormone in tissues of critically ill patients. *The Journal of Clinical Endocrinology & Metabolism*.

[49] Colombo P. C., Banchs J. E., Celaj S. (2005). Endothelial cell activation in patients with decompensated heart failure. *Circulation*.

[50] Colombo P. C., Onat D., Harxhi A. (2014). Peripheral venous congestion causes inflammation, neurohormonal, and endothelial cell activation. *European Heart Journal*.

[51] Nagaya T., Fujieda M., Otsuka G., Yang J.-P., Okamoto T., Seo H. (2000). A potential role of activated NF-*κ*B in the pathogenesis of euthyroid sick syndrome. *Journal of Clinical Investigation*.

[52] Wajner S. M., Goemann I. M., Bueno A. L., Larsen P. R., Maia A. L. (2011). IL-6 promotes nonthyroidal illness syndrome by blocking thyroxine activation while promoting thyroid hormone inactivation in human cells. *Journal of Clinical Investigation*.

[53] Hamilton M. A., Stevenson L. W., Luu M., Walden J. A. (1990). Altered thyroid hormone metabolism in advanced heart failure. *Journal of the American College of Cardiology*.

[54] Díez J. J., Iglesias P., Burman K. D. (2005). Spontaneous normalization of thyrotropin concentrations in patients with subclinical hypothyroidism. *The Journal of Clinical Endocrinology & Metabolism*.

[55] Pingitore A., Landi P., Taddei M. C., Ripoli A., L’Abbate A., Iervasi G. (2005). Triiodothyronine levels for risk stratification of patients with chronic heart failure. *The American Journal of Medicine*.

[56] Surks M. I., Chopra I. J., Mariash C. N., Nicoloff J. T., Solomon D. H. (1990). American thyroid association guidelines for use of laboratory tests in thyroid disorders. *JAMA: The Journal of the American Medical Association*.

[57] Gheorghiade M., Pang P. S. (2009). Acute heart failure syndromes. *Journal of the American College of Cardiology*.

