# Supplementary material for: Evaluation of Thyroid Function in Patients Hospitalized for Acute Heart Failure
Source: Int J Endocrinol. 2021 Mar 31;2021:6616681. doi: 10.1155/2021/6616681 (PMC8026290; doi:10.1155/2021/6616681)
Supplement: Supplementary Materials — Table S1. The values shown are linear regression coefficients (β) and 95% confidence intervals, estimated by uni- and multivariate linear regressions, with TSH or FT4 as the independent variable and heart rate, systolic BP, diastolic BP, BNP, LV ejection fraction, and PASP as dependent variables. Table S2. The values shown are linear regression coefficients (β) and 95% confidence intervals, estimated by uni- and multivariate linear regressions, with TSH or FT4 as the independent variable and inhospital mortality, hospital readmission, and all-cause and cardiovascular mortality within 30 days of discharge as dependent variables. [file 6616681.f1.zip › Supplementary Material.docx]

| **Supplementary Table S1. Linear association of thyroid function (****TSH and FT4) with cardiovascular function during hospitalization in euthyroid patients** | | | | | |
| --- | --- | --- | --- | --- | --- |
|  | **TSH (μIU/mL)** | |  | **FT4 (ng/dL)** | |
|  | **β (95% CI)** | **P value** |  | **β (95% CI)** | **P value** |
| **Heart rate**, bpm |  |  |  |  |  |
| Unadjusted | -2.31 (-8.08 to 3.46) | 0.431 |  | 7.36 (-7.81 to 22.53) | 0.339 |
| Model 1 ^a^ | -2.40 (-8.21 to 3.41) | 0.416 |  | 6.96 (-8.33 to 22.24) | 0.370 |
| **Blood pressure** |  |  |  |  |  |
| **Systolic BP**, mmHg |  |  |  |  |  |
| Unadjusted | 1.80 (-4.58 to 8.18) | 0.578 |  | -11.58 (-27.56 to 4.40) | 0.154 |
| Model 1 ^a^ | 1.15 (-5.15 to 7.45) | 0.719 |  | -10.09 (-26.04 to 5.87) | 0.214 |
| **Diastolic BP**, mmHg |  |  |  |  |  |
| Unadjusted | 3.89 (-0.02 to 7.81) | 0.051 |  | -3.93 (-14.15 to 6.30) | 0.449 |
| Model 1 ^a^ | 3.66 (-0.27 to 7.59) | 0.068 |  | -3.24 (-13.51 to 7.03) | 0.534 |
| **BNP,** pg/mL |  |  |  |  |  |
| Unadjusted model | 0.07 (-0.16 to 0.30) | 0.560 |  | 0.33 (-0.27 to 0.92) | 0.278 |
| Model 1 ^a^ | 0.11 (-0.11 to 0.34) | 0.320 |  | 0.24 (-0.34 to 0.81) | 0.421 |
| **Echocardiographic measures** |  |  |  |  |  |
| **LV ejection fraction,** % |  |  |  |  |  |
| Unadjusted model | -1.22 (-9.80 to 7.36) | 0.775 |  | -15.54 (-45.24 to 14.15) | 0.294 |
| Model 1 ^a^ | -1.02 (-9.49 to 7.45) | 0.808 |  | -14.43 (-44.48 to 15.62) | 0.334 |
| **PASP,** mmHg |  |  |  |  |  |
| Unadjusted model | 1.83 (-4.84 to 8.50) | 0.585 |  | 7.35 (-14.04 to 28.74) | 0.494 |
| Model 1 ^a^ | 1.82 (-4.95 to 8.59) | 0.593 |  | 8.88 (-13.00 to 30.77) | 0.419 |

The values shown are linear regression coefficients (β) and 95% confidence intervals, estimated by uni- and multivariate linear regressions, with TSH or FT4 as the independent variable and heart rate, systolic BP, diastolic BP, BNP, LV ejection fraction and PASP as dependent variables. TSH and BNP were log-transformed. TSH, thyroid-stimulating hormone; FT4, free thyroxine; CI, confidence interval; BP, blood pressure; BNP, B-type natriuretic peptide; LV, left ventricle; PASP: pulmonary arterial systolic pressure ^a^ Adjusted for age and sex.

| **Supplementary Table S2. Association of thyroid function (TSH and FT4) with short-term clinical endpoints in euthyroid patients** | | | | | |
| --- | --- | --- | --- | --- | --- |
|  | **TSH** (μIU/mL) | |  | **FT4** (ng/dL) | |
|  | **OR (95% CI)** | **P Value** |  | **OR (95% CI)** | **P Value** |
| **In-hospital mortality** |  |  |  |  |  |
| Unadjusted model | 0.75 (0.25 to 2.24) | 0.610 |  | 0.39 (0.01 to 21.34) | 0.644 |
| Model 1 ^a^ | 0.77 (0.26 to 2.32) | 0.646 |  | 0.36 (0.01 to 21.29) | 0.621 |
| **Readmission for heart failure within 30 days** |  |  |  |  |  |
| Unadjusted model | 1.02 (0.61 to 1.69) | 0.950 |  | 3.43 (0.91 to 12.86) | 0.068 |
| Model 1 ^a^ | 1.03 (0.62 to 1.73) | 0.900 |  | 3.26 (0.85 to 12.51) | 0.085 |
| **All-cause mortality within 30 days** |  |  |  |  |  |
| Unadjusted model | 0.67 (0.34 to 1.31) | 0.243 |  | **4.34 (1.05 to 17.88)** | **0.042** |
| Model 1 ^a^ | 0.67 (0.34 to 1.33) | 0.253 |  | **4.40 (1.06 to 18.16)** | **0.041** |
| **Cardiovascular mortality within 30 days** |  |  |  |  |  |
| Unadjusted model | 1.08 (0.47 to 2.48) | 0.857 |  | **6.63 (1.39 to 31.70)** | **0.018** |
| Model 1 ^a^ | 1.09 (0.47 to 2.54) | 0.841 |  | **6.92 (1.34 to 35.70)** | **0.021** |

The values shown are linear regression coefficients (β) and 95% confidence intervals, estimated by uni- and multivariate linear regressions, with TSH or FT4 as the independent variable and in-hospital mortality, hospital readmission and all-cause and cardiovascular mortality within 30 days of discharge as dependent variables. TSH was log-transformed. TSH, thyroid-stimulating hormone; FT4, free thyroxine; CI, confidence interval. ^a^ Adjusted for age and sex.

| **Supplementary Table S3. Association between inpatient parameters and long-term clinical outcomes in euthyroid patients** | | | | | |
| --- | --- | --- | --- | --- | --- |
|  | **Unadjusted model** | |  | **Age- and sex-adjusted model** | |
|  | **Hazard ratio** (95% CI) | **P Value** |  | **Hazard ratio (**95% CI) | **P Value** |
| **All-cause mortality** | | | | | |
| TSH (μIU/mL) | 0.98 (0.75 to 1.28) | 0.892 |  | 0.97 (0.74 to 1.27) | 0.843 |
| FT4 (ng/dL) | 1.44 (0.66 to 3.12) | 0.361 |  | 1.19 (0.54 to 2.63) | 0.664 |
| BNP (pg/mL) | **1.22 (1.03 to 1.44)** | **0.018** |  | **1.23 (1.04 to 1.45)** | **0.018** |
| eGFR | **0.99 (0.98 to 1.00)** | **0.003** |  | **0.99 (0.98 to 1.00)** | **0.022** |
| LV ejection fraction (%) | 0.98 (0.95 to 1.01) | 0.125 |  | 0.98 (0.96 to 1.01) | 0.172 |
| PASP (mmHg) | **1.03 (1.02 to 1.05)** | **<0.001** |  | **1.04 (1.02 to 1.05)** | **<0.001** |
| **Cardiovascular mortality** | | | | | |
| TSH (μIU/mL) | 0.99 (0.68 to 1.43) | 0.961 |  | 0.98 (0.67 to 1.42) | 0.914 |
| FT4 (ng/dL) | 1.09 (0.34 to 3.49) | 0.881 |  | 1.04 (0.32 to 3.41) | 0.946 |
| BNP (pg/mL) | **1.41 (1.11 to 1.79)** | **0.005** |  | **1.44 (1.13 to 1.83)** | **0.003** |
| eGFR (mL/min/1.73m^2^) | 0.99 (0.98 to 1.00) | 0.229 |  | 0.99 (0.98 to 1.00) | 0.269 |
| LV ejection fraction (%) | 0.98 (0.95 to 1.02) | 0.350 |  | 0.98 (0.94 to 1.02) | 0.310 |
| PASP (mmHg) | **1.04 (1.02 to 1.06)** | **<0.001** |  | **1.04 (1.02 to 1.06)** | **<0.001** |

TSH and BNP were log-transformed. CI, confidence interval; TSH, thyroid-stimulating hormone; FT4, free thyroxine; BNP, B-type natriuretic peptide; eGFR, estimated glomerular filtration rate; LV, left ventricle; PASP, pulmonary arterial systolic pressure.
