# Supplementary material for: Evaluation of Thyroid Function in Patients Hospitalized for Acute Heart Failure
Source: Int J Endocrinol. 2021 Mar 31;2021:6616681. doi: 10.1155/2021/6616681 (PMC8026290; doi:10.1155/2021/6616681)
Supplement: Supplementary Materials — Table S1. The values shown are linear regression coefficients (β) and 95% confidence intervals, estimated by uni- and multivariate linear regressions, with TSH or FT4 as the independent variable and heart rate, systolic BP, diastolic BP, BNP, LV ejection fraction, and PASP as dependent variables. Table S2. The values shown are linear regression coefficients (β) and 95% confidence intervals, estimated by uni- and multivariate linear regressions, with TSH or FT4 as the independent variable and inhospital mortality, hospital readmission, and all-cause and cardiovascular mortality within 30 days of discharge as dependent variables. [file 6616681.f1.zip › Strobe Statement.doc]

STROBE Statement—checklist of items that should be included in reports of observational studies

|  | Item No | Recommendation |
| --- | --- | --- |
| **Title and abstract** | 1 | (*a*) Indicate the study’s design with a commonly used term in the title or the abstract **Pages 1-2** |
|  |  | (*b*) Provide in the abstract an informative and balanced summary of what was done and what was found  **Page 2** |
| Introduction | | |
| Background/rationale | 2 | Explain the scientific background and rationale for the investigation being reported  **Pages 3-4** |
| Objectives | 3 | State specific objectives, including any prespecified hypotheses  **Page 4 (lines 86 to 94)** |
| Methods | | |
| Study design | 4 | Present key elements of study design early in the paper  **Pages 4-5** |
| Setting | 5 | Describe the setting, locations, and relevant dates, including periods of recruitment, exposure, follow-up, and data collection  **Pages 4-7** |
| Participants | 6 | (*a*) *Cohort study*—Give the eligibility criteria, and the sources and methods of selection of participants. Describe methods of follow-up  **Pages 4-6**  *Case-control study*—Give the eligibility criteria, and the sources and methods of case ascertainment and control selection. Give the rationale for the choice of cases and controls  *Cross-sectional study*—Give the eligibility criteria, and the sources and methods of selection of participants |
|  |  | (*b*) *Cohort study*—For matched studies, give matching criteria and number of exposed and unexposed  **Not Applicable**  *Case-control study*—For matched studies, give matching criteria and the number of controls per case |
| Variables | 7 | Clearly define all outcomes, exposures, predictors, potential confounders, and effect modifiers. Give diagnostic criteria, if applicable  **Pages 5-7** |
| Data sources/ measurement | 8* | For each variable of interest, give sources of data and details of methods of assessment (measurement). Describe comparability of assessment methods if there is more than one group  **Pages 5, 6 (lines 111 to 144)** |
| Bias | 9 | Describe any efforts to address potential sources of bias  **Page 7 (lines 162 to 171)** |
| Study size | 10 | Explain how the study size was arrived at  **Pages 4-5 (lines 98 to 107)** |
| Quantitative variables | 11 | Explain how quantitative variables were handled in the analyses. If applicable, describe which groupings were chosen and why  **Page 7 (lines 158 to 174)** |
| Statistical methods | 12 | (*a*) Describe all statistical methods, including those used to control for confounding  **Page 7 (lines 158 to 174)** |
|  |  | (*b*) Describe any methods used to examine subgroups and interactions  **Page 7 (lines 160 to 161)** |
|  |  | (*c*) Explain how missing data were addressed  **Page 8 (lines 179 to 185)** |
|  |  | (*d*) *Cohort study*—If applicable, explain how loss to follow-up was addressed  **Page 6 (lines 146 to 149)**  *Case-control study*—If applicable, explain how matching of cases and controls was addressed  *Cross-sectional study*—If applicable, describe analytical methods taking account of sampling strategy |
|  |  | (*e*) Describe any sensitivity analyses  **Page 7 (lines 171-172)** |

Continued on next page

| Results | | |
| --- | --- | --- |
| Participants | 13* | (a) Report numbers of individuals at each stage of study—eg numbers potentially eligible, examined for eligibility, confirmed eligible, included in the study, completing follow-up, and analysed  **Page 5, 6 (lines 98 to 107)** |
|  |  | (b) Give reasons for non-participation at each stage  **Not Applicable** |
|  |  | (c) Consider use of a flow diagram  **Not Applicable** |
| Descriptive data | 14* | (a) Give characteristics of study participants (eg demographic, clinical, social) and information on exposures and potential confounders  **Page 8 (lines 186 to 192)** |
|  |  | (b) Indicate number of participants with missing data for each variable of interest  **Page 8 (lines 179 to 185)** |
|  |  | (c) *Cohort study*—Summarise follow-up time (eg, average and total amount)  **Page 10 (line 226)** |
| Outcome data | 15* | *Cohort study*—Report numbers of outcome events or summary measures over time  **Pages 9, 10 (lines 210 to 212 and 226 to 229)** |
|  |  | *Case-control study—*Report numbers in each exposure category, or summary measures of exposure |
|  |  | *Cross-sectional study—*Report numbers of outcome events or summary measures |
| Main results | 16 | (*a*) Give unadjusted estimates and, if applicable, confounder-adjusted estimates and their precision (eg, 95% confidence interval). Make clear which confounders were adjusted for and why they were included  **Pages 8-10** |
|  |  | (*b*) Report category boundaries when continuous variables were categorized  **Not applicable** |
|  |  | (*c*) If relevant, consider translating estimates of relative risk into absolute risk for a meaningful time period  **Not applicable** |
| Other analyses | 17 | Report other analyses done—eg analyses of subgroups and interactions, and sensitivity analyses  **Pages 9-10 + Supplementary Material** |
| Discussion | | |
| Key results | 18 | Summarise key results with reference to study objectives  **Pages 10-11 (lines 244 to 252)** |
| Limitations | 19 | Discuss limitations of the study, taking into account sources of potential bias or imprecision. Discuss both direction and magnitude of any potential bias  **Page 14 (lines 337 to 350)** |
| Interpretation | 20 | Give a cautious overall interpretation of results considering objectives, limitations, multiplicity of analyses, results from similar studies, and other relevant evidence  **Pages 10-15** |
| Generalisability | 21 | Discuss the generalisability (external validity) of the study results  **Page 15 (lines 351 to 364)** |
| Other information | | |
| Funding | 22 | Give the source of funding and the role of the funders for the present study and, if applicable, for the original study on which the present article is based  **Page 16 (lines 382 to 389)** |

*Give information separately for cases and controls in case-control studies and, if applicable, for exposed and unexposed groups in cohort and cross-sectional studies.

**Note:** An Explanation and Elaboration article discusses each checklist item and gives methodological background and published examples of transparent reporting. The STROBE checklist is best used in conjunction with this article (freely available on the Web sites of PLoS Medicine at http://www.plosmedicine.org/, Annals of Internal Medicine at http://www.annals.org/, and Epidemiology at http://www.epidem.com/). Information on the STROBE Initiative is available at www.strobe-statement.org.
